# Development and validation of a nutritional risk prediction model for patients with ulcerative colitis: a single-center retrospective study

**DOI:** 10.1038/s41598-025-33379-8

**Published:** 2025-12-29

**Authors:** Xiaorong Yang, Yunhui Zhang, Xieqiao He, Rong Liu, Mengxia Li, Haiyan Zhang

**Affiliations:** 1 Faculty of Nursing, Kunming Medical University, Kunming, 650500 Yunan China; 2 First Affiliated Hospital of Kunming Medical University, Kunming, 650032 Yunan China

**Keywords:** Ulcerative colitis, Nutritional risk, Prediction model, Risk stratification, Biomarkers, Diseases, Gastroenterology, Medical research, Risk factors

## Abstract

Nutritional risk is commonly overlooked in patients with ulcerative colitis (UC) due to a lack of specific assessment tools. This study developed and validated a prediction model to identify nutritional risk using routine clinical parameters. Conducted at a tertiary hospital in China, this retrospective analysis utilized data from 451 UC patients between January 2016 and October 2023, with temporal validation in a cohort of 115 patients from November 2023 to September 2024. Through multivariable logistic regression, we identified five independent predictors: diet, gender, disease activity, albumin levels, and hemoglobin levels, and created a nomogram. The model demonstrated strong performance, achieving an area under the ROC curve (AUC) of 0.914 in internal validation and 0.875 in temporal validation. Calibration curves showed good agreement between predicted and observed outcomes, and DCA confirmed clinical utility across different risk thresholds. In conclusion, our nomogram offers clinicians a practical tool for early nutritional intervention in UC, potentially reducing long-term complications. However, as a preliminary model derived from a single center, it requires further multi-center external validation before widespread clinical application.

**Supplementary Information:**

The online version contains supplementary material available at 10.1038/s41598-025-33379-8.

## Introduction

Ulcerative colitis (UC) is a chronic, progressive, and potentially destructive condition that cannot be completely cured^1^. It can lead to long-term intestinal impairment and a loss of function. UC is a subtype of inflammatory bowel disease (IBD)^2^. Nutritional risk is common among hospitalized UC patients and is associated with poor long-term outcomes^3,4^. Several studies have shown that UC patients are at high nutritional risk^5–8^. Consequently, current clinical practice guidelines recommend screening patients for nutritional risk at the time of diagnosis and regularly thereafter^9^. Early screening of patients’ nutritional status and identification of potential risks can effectively prevent adverse outcomes.

Nutritional risk assessments commonly utilize screening tools such as the Nutritional Risk Screening 2002 (NRS-2002), the Malnutrition Universal Screening Tool (MUST), and the Malnutrition Screening Tool (MST), along with IBD-specific instruments like the Malnutrition Inflammation Risk Tool (MIRT) and the Saskatchewan IBD-Nutrition Risk (SASKIBD-NR)^10^. However, the lack of a standardized tool specifically for UC creates two major issues: (1) it hinders valid cross-study prevalence comparisons due to the use of heterogeneous assessment methods, and (2) it leads to inconsistent clinical recommendations and monitoring practices arising from tool-dependent risk classifications. These limitations impede targeted nutritional interventions for UC populations.

To address this unmet need, we aimed to develop a prediction model that estimates the probability of a patient being at nutritional risk (as defined by NRS-2002 ≥ 3) using only routinely available clinical and laboratory data. This approach does not seek to replace NRS-2002, but to enable proactive, early identification of at-risk individuals based on data already in the electronic medical record (EMR), potentially before a formal NRS-2002 screening is conducted or in settings where it is not routinely performed. This could help streamline clinical workflows for gastroenterologists and nurses.

## Materials and methods

### Ethical issue

This single-center, retrospective cohort study was conducted at the First Affiliated Hospital of Kunming Medical University in Yunnan, China, between January 2016 and September 2024. The study adhered to the Transparent Reporting of a Multivariate Prediction Model for Individual Prognosis or Diagnosis (TRIPOD) statement as a reporting guideline^11^. It was approved by the Ethics Committee of the First Affiliated Hospital of Kunming Medical University (Approval Number: 2024-L-058). All methods were performed in accordance with the relevant guidelines and regulations. The requirement for informed consent was waived by the Ethics Committee due to the retrospective nature of the study and the anonymization of all patient data.

### Study population

**Fig. 1:**
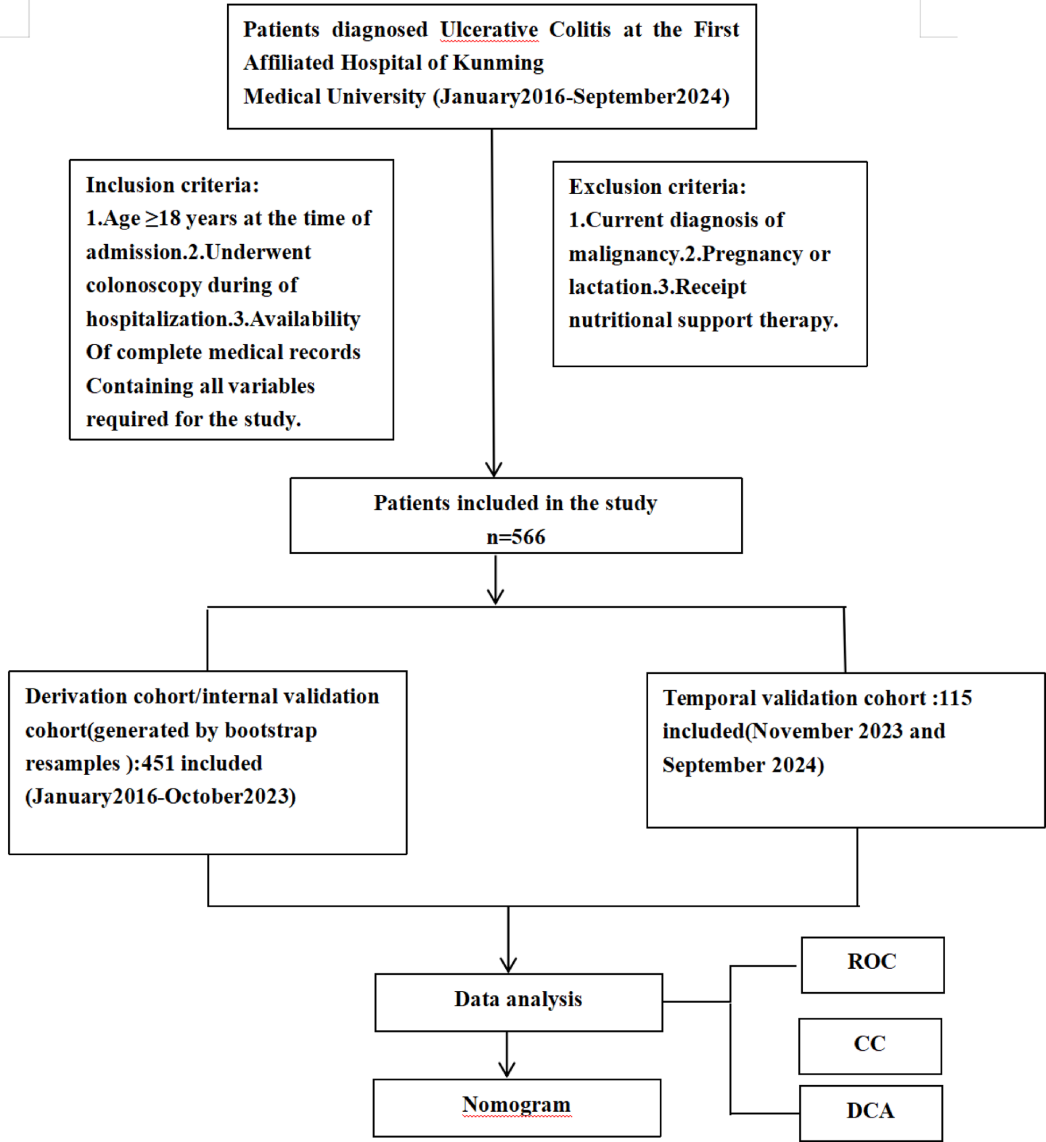
Patient selection flow chart for the derivation and temporal validation cohorts.

This study consecutively enrolled hospitalized patients diagnosed with UC at The First Affiliated Hospital of Kunming Medical University (see Fig. 1). The model development and internal validation cohort included patients admitted between January 2016 and October 2023 (*n* = 451). For external temporal validation, an independent cohort of patients admitted between November 2023 and September 2024 (*n* = 115) was retrospectively collected from the same institution. Eligible patients were included based on the following criteria: hospitalized patients diagnosed with UC according to the 2018 consensus guidelines on the diagnosis and management of IBD established by the IBD Group of the Chinese Society of Gastroenterology, Chinese Medical Association^12^; Aged 18 years or older at the time of admission; Underwent a colonoscopy during hospitalization.; Availability of complete medical records containing all required variables for the study. Exclusion criteria were: Concurrent diagnosis of malignancy; Pregnancy or lactation; Receipt of nutritional support therapy at the time of admission.

### Sample size calculation

The sample size was determined using the Events Per Variable (EPV) principle. With 129 outcome events related to nutritional risk and 5 predictors in the final model, the EPV calculated was 25.8. This exceeds the recommended minimum threshold of EPV ≥ 10, ensuring that there is adequate statistical power for model development.

### Outcome variables

The outcome of the study was nutritional risk, defined as a hospitalised patient with a Nutritional Risk Screening (NRS)-2002 score ≥ 3. The (NRS)-2002 was developed by the European Society for Parenteral and Enteral Nutrition (ESPEN) in 2002 to streamline nutritional risk screening for hospitalised patients^13^. Since then, it has been widely adopted by the Chinese Society of Parenteral and Enteral Nutrition and the Chinese Medical Association for nutritional risk assessment^14,15^. The scale consists of three parts: disease status (0–3 points), nutritional status (0–3 points), and age score (0–1 points), and the sum of the three items is the total score, and the total score ≥ 3 points suggests that the patient is at nutritional risk, and the higher the total score of NRS2002, the higher the nutritional risk. The NRS-2002 assessment was routinely performed by trained ward nurses within 24 h of patient admission, as part of the standard nursing assessment protocol. Data were extracted from this standardized section of the electronic medical record.

### Candidate variables

Through a comprehensive review of the literature^16–19^ and integration of clinical expertise, a total of 19 candidate variables were selected for further analysis. The collected variables included: (1)Demographic and baseline characteristics: age, sex, educational level, contact information, marital status, length of hospital stay, medical payment method, BMI, smoking history, alcohol consumption, sleep patterns, and dietary habits (Dietary intake was categorized as ‘normal’ or ‘abnormal’ based on retrospective review of physician and nursing notes in the electronic medical record. ‘Normal diet’ was defined as documentation indicating the patient was consuming a regular or general diet without significant restrictions or reports of poor intake. ‘Abnormal diet’ included any documented dietary modifications (e.g., liquid, soft, or low-residue diets), patient-reported anorexia, or notes indicating intake was less than 50% of usual); (2)Disease-related factors: disease activity (disease severity was assessed using Mayo clinic score in patients with UC, active disease was defined as Mayo clinic score ≥ 3 for UC), Lesion extent, type of lesion, disease duration, age at diagnosis, treatment regimens (medications/surgery), history of diabetes, prior gastrointestinal surgeries, and complications; (3)Laboratory parameters: erythrocyte sedimentation rate (ESR), C-reactive protein (CRP), hemoglobin (HB), and serum albumin levels (ALB).

### Data collection

Data were collected retrospectively from the hospital’s electronic medical record (EMR) system using a structured questionnaire that covered three specific domains. The collection process strictly followed predefined inclusion and exclusion criteria. It involved real-time data entry and rigorous quality control measures, including dual-entry verification and prompt cross-checking against source documents to identify and resolve discrepancies or missing values. To ensure methodological rigor and the reliability of the results, a professional statistician supervised the statistical analysis process.

### Model derivation

Potential predictors of nutritional risk were initially identified through univariate analysis using SAS software. Variables with a P-value of less than 0.10 from the univariate comparisons (utilizing either the Chi-square test or Mann-Whitney U test, as appropriate) were selected for further analysis. These variables were then included in a binary logistic regression model in SAS, where the presence or absence of nutritional risk acted as the dependent variable. Backward stepwise selection, based on the likelihood ratio criterion, was used to identify independent risk factors. The final multivariable model was checked for multicollinearity using variance inflation factors (VIF < 5). The predictors retained from the logistic regression analysis were used to create a nutritional risk prediction model for patients with ulcerative colitis (UC) using R software. Based on the final multivariable logistic regression model, a static nomogram was developed using the rms package in R version 4.3.3. Each predictor was assigned a point score that corresponds proportionally to its regression coefficient from the logistic model. The total point score indicates the predicted probability of nutritional risk, enabling clinical estimations without relying on computational tools.

### Prediction model performance

The fixed coefficients from a multivariable logistic regression model, derived from a cohort of 451 subjects, were applied to an internal validation set (1,000 bootstrap resamples) and a temporal validation cohort (*n* = 115). The model was evaluated for discrimination using the AUC, for calibration with the Hosmer-Lemeshow test and calibration plots, and for clinical utility via decision curve analysis in the derivation set. All statistical analyses were performed using SAS 9.4 and R 4.3.3.

## Results

### Characteristics of the cohorts

The derivation cohort consisted of 451 UC patients hospitalized between January 2016 and October 2023, while the temporal validation cohort comprised 115 patients admitted from November 2023 to September 2024. Nutritional risk prevalence was comparable between cohorts, occurring in 129 patients (28.6%) in the derivation group and 30 patients (26.1%) in the temporal validation group. With no significant differences observed between the two groups regarding.

gender, age, disease duration, smoking or alcohol consumption status, treatment history, diabetes status, sleep patterns, dietary habits, BMI distribution, education level, disease activity, lesion type, lesion extent, ESR, CRP, Hb levels, presence of complications, or history of gastrointestinal diseases. This comparability minimized potential bias arising from uneven distribution (*P* > 0.05). However, statistically significant differences were found in hospitalization duration, marital status, medical payment method composition, and whether ALB levels were normal (all *P* < 0.05), as detailed in Table 1.

**Table 1:** Baseline characteristics of patients in the derivation and Temporal validation cohorts.

Variables	Derivation cohort *N* = 451(%)	Validation cohort *N* = 115 (%)	*P*-value
Nutritional risk			
Yes	129 (28.60)	30 (26.09)	0.582
No	322 (71.40)	85 (73.91)	
Gender			
Male	275 (60.98)	66 (57.39)	0.483
Female	176 (39.02)	49 (42.61)	
Length of hospital stay (days)			
≤ 8	274 (60.75)	82 (71.30)	0.037
> 8	177 (39.25)	33 (28.70)	
Age at onset (years)			
≤ 16	2 (0.44)	1 (0.87)	0.190
17 ~ 40	206 (45.68)	42 (36.52)	
> 40	243 (53.88)	72 (62.61)	
Marital status			
Unmarried	37 (8.20)	7 (6.09)	<0.001
Married	408 (90.47)	99 (86.09)	
Other	6 (1.33)	9 (7.82)	
Medical payment method			
Urban Employee	140 (31.04)	50 (43.48)	<0.001
Urban resident	224 (49.67)	60 (52.17)	
Other	87 (19.29)	5 (4.35)	
Disease duration (years)			
≤ 5	308 (68.29)	71 (61.74)	0.100
6 ~ 10	85 (18.85)	32 (27.83)	
>10	58 (12.86)	12 (10.43)	
Smoking history			
No	390 (86.47)	97 (84.35)	0.557
Yes	61 (13.53)	18 (15.65)	
History of alcoholism			
No	415 (92.02)	105 (91.30)	0.803
Yes	36 (7.98)	10 (8.70)	
Treatment regimen			
Monotherapy	364 (80.71)	101 (87.83)	0.075
Polytherapy	87 (19.29)	14 (12.17)	
Diabetes			
No	435 (96.45)	108 (93.91)	0.218
Yes	16 (3.55)	7 (6.09)	
Sleep quality			
Normal	311 (68.96)	74 (64.35)	0.344
Abnormal	140 (31.04)	41 (35.65)	
Dietary			
Normal	319 (70.73)	90 (78.26)	0.107
Abnormal	132 (29.27)	25 (21.74)	
BMI			
Underweight	88 (19.52)	22 (19.13)	0.738
Normal	267 (59.20)	63 (54.78)	
Overweight	77 (17.07)	24 (20.87)	
Obese	19 (4.21)	6 (5.22)	
Educational level			
Primary and below	148 (32.82)	37 (32.17)	0.409
Middle and high school	173 (38.36)	38 (33.04)	
College and above	130 (28.82)	40 (34.79)	
Disease activity			
Remission period	52 (11.53)	8 (6.95)	0.121
Mild	73 (16.18)	25 (21.74)	
Moderate	184 (40.80)	54 (46.96)	
Severe	142 (31.49)	28 (24.35)	
Type of lesion			
Initial onset	25 (5.54)	6 (5.22)	0.891
Chronic relapsing type	426 (94.46)	109 (94.78)	
Extent of Lesion			
Rectal type	89 (19.73)	15 (13.05)	0.218
Left-sided colonic type	134 (29.72)	40 (34.78)	
Extensive colonic type	228 (50.55)	60 (52.17)	
Albumin level			
Normal	214 (47.45)	74 (64.35)	0.001
Abnormal	237 (52.55)	41 (35.65)	
Erythrocyte sedimentation rate			
Normal	97 (21.51)	23 (20.00)	0.724
Abnormal	354 (78.49)	92 (80.00)	
C-reactive protein			
Normal	282 (62.53)	80 (69.57)	0.161
Abnormal	169 (37.47)	35 (30.43)	
Hemoglobin level			
Normal	117 (25.94)	37 (32.17)	0.180
Abnormal	334 (74.06)	78 (67.83)	
Complications			
No	436 (96.67)	113 (98.26)	0.374
Yes	15 (3.33)	2 (1.74)	
History of gastrointestinal surgery			
No	408 (90.47)	102 (88.70)	0.570
Yes	43 (9.53)	13 (11.30)	

### Variable selection

#### Univariate and multivariate analysis

The derivation cohort consisted of 451 UC patients hospitalized between. Univariate analysis revealed significant associations between nutritional risk and disease duration, hospitalization length, treatment regimen, diabetes, sleep quality, diet, BMI, disease activity/extent, albumin, ESR, CRP, and HB (all *P* < 0.05). No associations were found with gender, age at onset, marital status, payment method, smoking, alcohol use, educational level, complications, or gastrointestinal surgery history (all *P* > 0.05; Supplemental Table 1).

Variables with *P* < 0.05 in univariate analysis (including sex, marital status, BMI, age, disease duration, smoking, alcohol use, treatment, and sleep) were entered into a backward stepwise logistic regression model (entry: *P* < 0.05; removal: *P* ≥ 0.10). No multicollinearity was detected (tolerance > 0.1; Variance Inflation Factor (VIF)<10; Supplemental Table 2). The final model identified five independent predictors (Table 2). CRP and ESR were significant in univariate analysis (*p* < 0.05) but were excluded in backward stepwise regression because they showed high multicollinearity with disease activity (Mayo score: VIF = 8.7 for CRP, VIF = 9.2 for ESR). Including these markers would have violated the assumption of no multicollinearity (VIF < 5). Hemoglobin was retained in the final model due to its strong clinical relevance and established pathophysiological link to nutritional status in UC (e.g., chronic blood loss, malabsorption), and because sensitivity analyses confirmed its consistent contribution to the model’s discriminative performance.

**Table 2:** Logistic regression analysis of factors associated with nutritional risk in UC patients (derivation cohort).

Variable	β	SE	Wald χ²	*P*-value	OR (95%CI)
Dietary	0.901	0.320	7.913	0.004	2.462 (1.314, 4.611)
Gender	1.196	0.452	7.009	0.008	3.306 (1.364, 8.013)
Disease activity	2.889	0.329	76.769	< 0.001	17.978 (9.420, 34.311)
Albumin	1.117	0.377	8.769	0.003	3.054 (1.459, 6.395)
Hemoglobin	0.979	0.531	3.399	0.065	2.662 (0.940, 7.538)

### Nomogram for clinical risk stratification

**Fig. 2:**
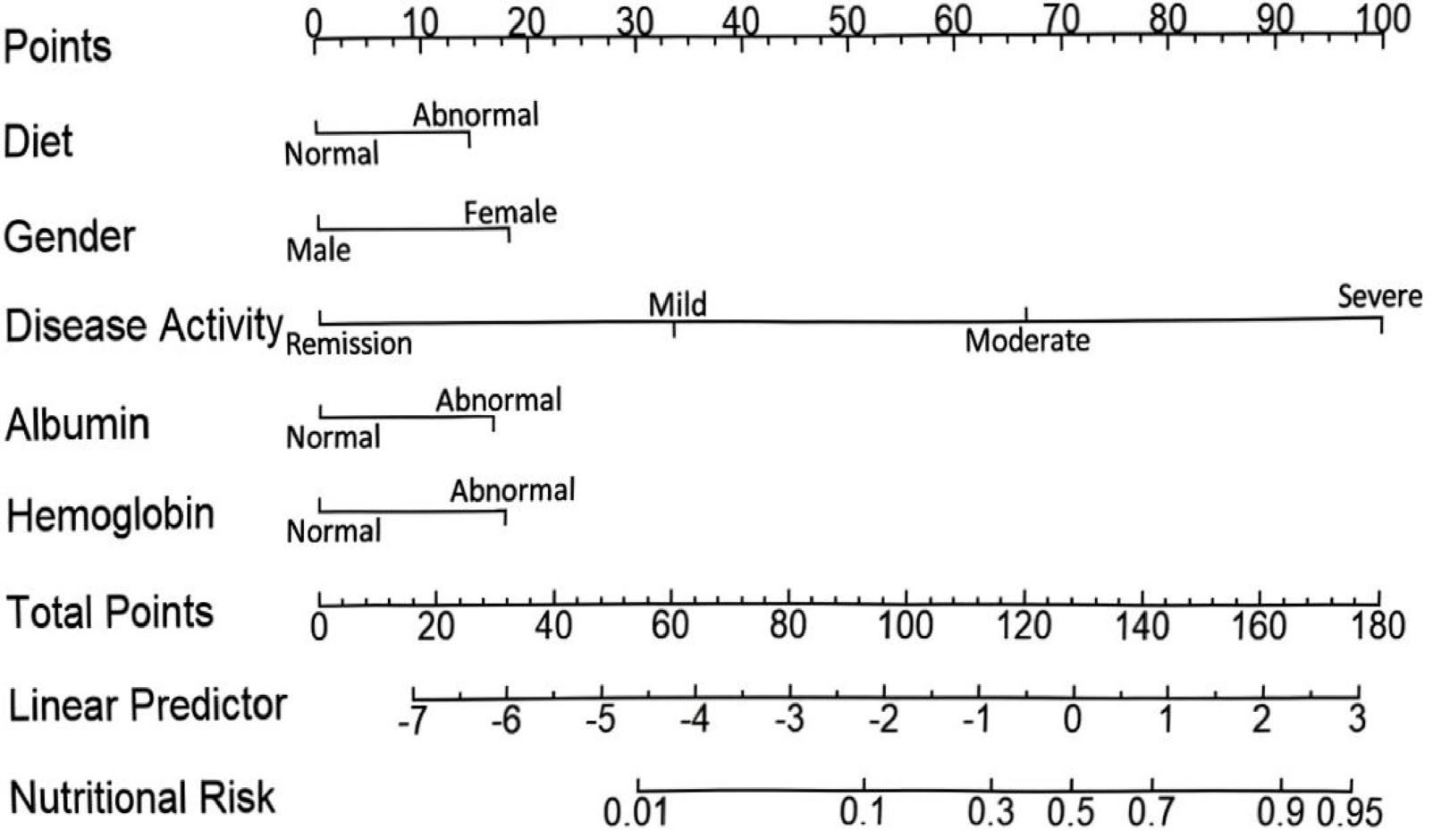
Static nomogram for predicting nutritional risk in patients with ulcerative colitis.

Logistic regression model results indicated that diet, gender, disease activity, albumin, and hemoglobin could serve as predictors of nutritional risk. Using the rms package, a Nomogram model was constructed to predict the occurrence of nutritional risk in UC patients, with the results visualized in Fig. 2. For example, a UC patient presenting with abnormal diet (23 points), male sex (0 points), severe disease activity (100 points), normal albumin (0 points), and abnormal hemoglobin (25 points) achieves a total score of 148 points. The predicted probability of nutritional risk for this patient is approximately 53%. This probability indicates the patient is at moderate nutritional risk; the patient does not require urgent nutritional intervention (reserved for probabilities ≥ 70%, corresponding to a total score ≥ 180), but should undergo further targeted assessment to confirm nutritional status and guide preventive interventions. This threshold is based on our DCA results, which show the model’s net benefit is highest for moderate-risk patients (50–70% probability) when combined with secondary assessments, balancing the need to avoid over-intervention and missed risks. To facilitate the clinical application and dissemination of the model, an interactive web-based dynamic Nomogram was developed. A Chinese version of the app is accessible at: https://liexiantukyxjcy.shinyapps.io/DynNomapp/. To reach a wider international audience, an English version has also been developed and is available at: https://eloise-apps.shinyapps.io/uc_nutrition_risk_english/.

### Prediction model performance in the internal validation cohort

#### Model calibration

**Fig. 3:**
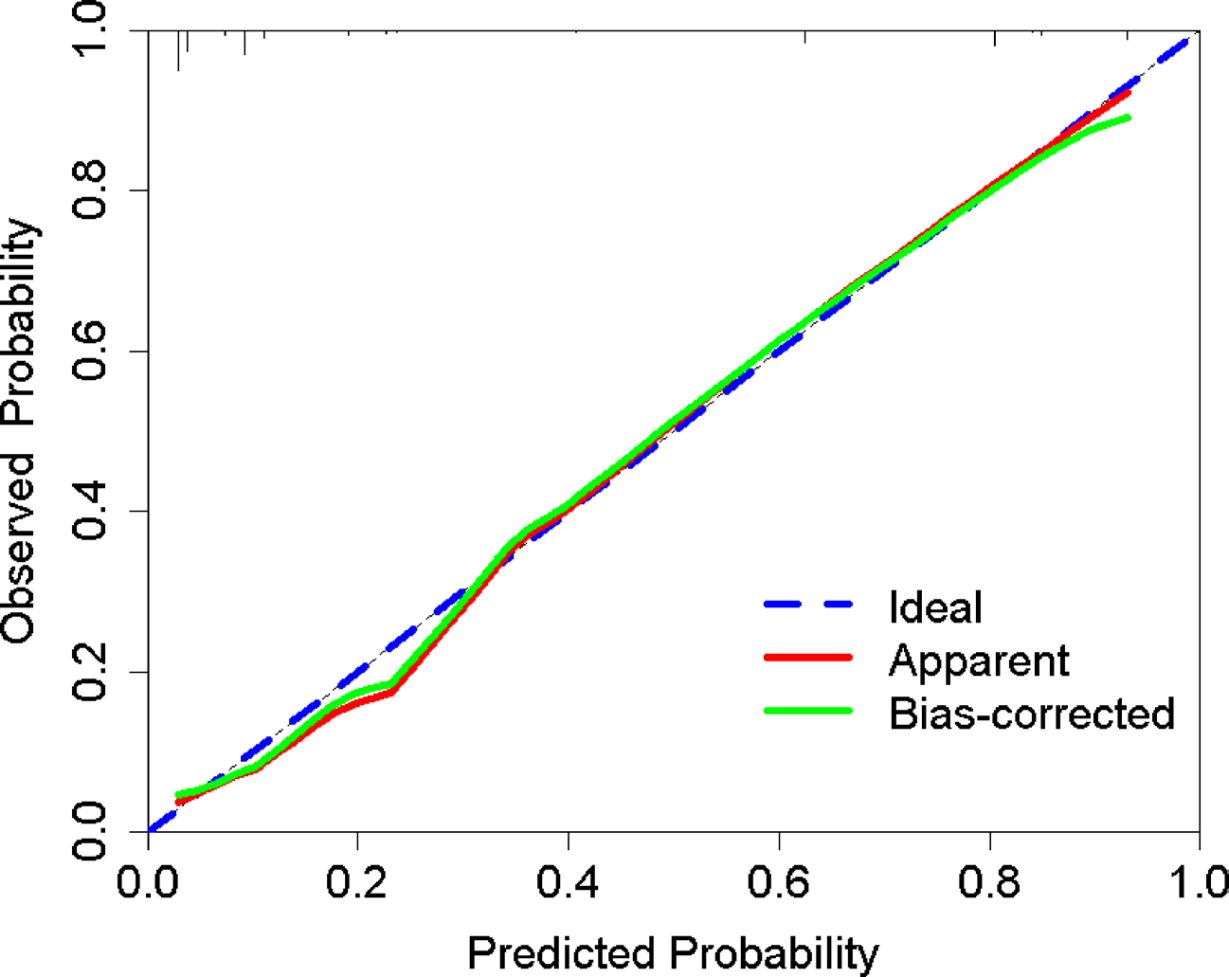
Calibration curve of the nutritional risk prediction model in the derivation cohort.

In the derivation cohort, the model demonstrated excellent calibration: a Brier score of 0.093 (< 0.25 threshold), a non-significant Hosmer-Lemeshow test (*P* = 0.902), and calibration curves showing close alignment between predicted and observed outcomes across risk strata (Supplemental Fig. 1). Bootstrap-corrected calibration (1000 iterations; Fig. 3) further confirmed good agreement, with the corrected curve (solid green) closely tracking the ideal reference line (dashed blue).

#### Model discrimination

**Fig. 4:**
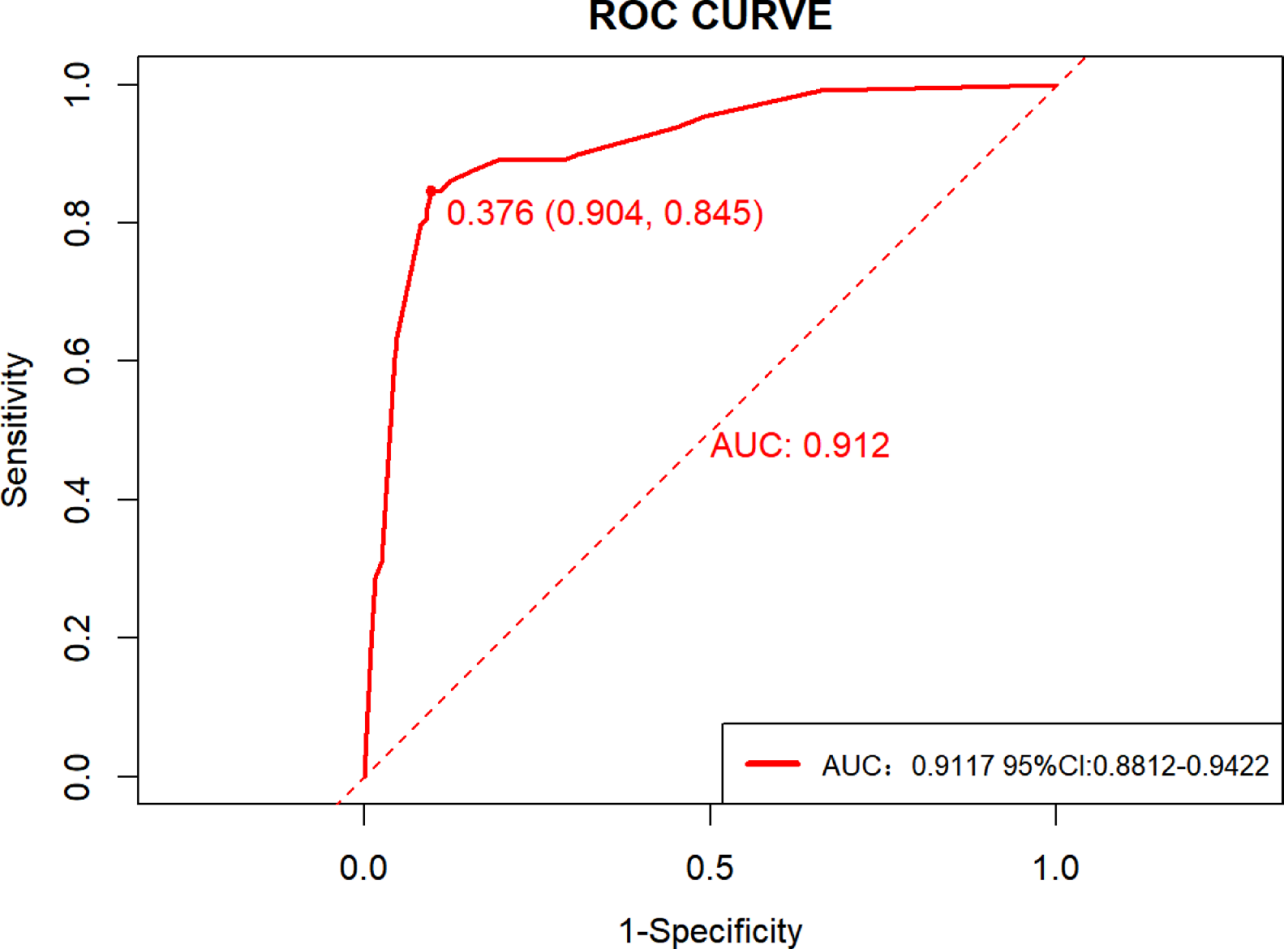
Receiver operating characteristic (ROC) curve of the model in the derivation cohort (AUC = 0.912, 95% CI: 0.881–0.942).

The ROC curve analysis conducted using the pROC package demonstrated that the prediction model for predicting nutritional risk in UC patients achieved an AUC of 0.9117 (95% CI: 0.8812–0.9422) in the Derivation cohort(Fig. 4). The optimal risk cut-off value was 0.376, corresponding to a specificity of 84.5%, a sensitivity of 90.4%, and a Youden’s index of 0.7487. At this threshold, the accuracy was 0.8869 and the precision (positive predictive value) was 0.7786. The confusion matrix is presented in Supplemental Table 3. Internal validation was performed using the bootstrap resampling method with 1000 replicates. The mean AUC derived from the validation ROC curve was 0.914 (95% CI: 0.880–0.942), as illustrated in Supplemental Fig. 2.

#### Clinical utility

**Fig. 5:**
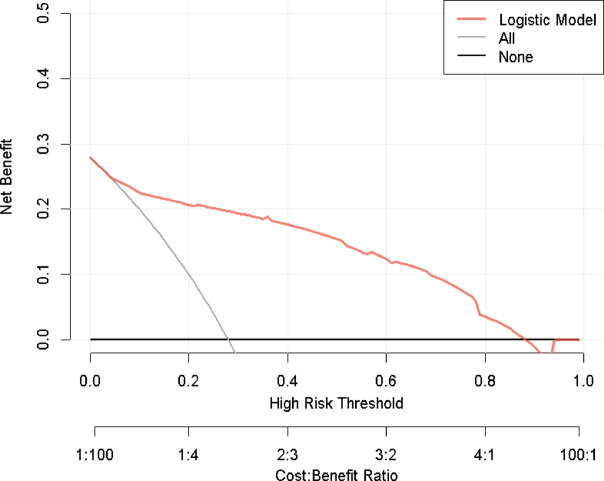
Decision curve analysis (DCA) of the model in the derivation cohort, showing a net benefit across a wide range of threshold probabilities.

Clinical decision curve analysis (DCA) was performed using the rmda package. The DCA curve circumvents the need to explicitly consider false positives and false negatives by directly calculating the net benefit (NB), thereby maximizing it. The DCA plot displays the threshold probability on the x-axis and the net benefit (NB) on the y-axis. The horizontal reference line signifies the strategy of “treat none” (considering all samples negative and with holding intervention for all patients), where the net benefit is zero. The straight diagonal reference line represents the strategy of “treat all” (considering all samples positive and providing intervention to all patients), characterized by a negative slope. As shown in Fig. 5, the DCA curve (red line) demonstrates a net benefit exceeding those of both the “treat none” (black horizontal line) and “treat all” (light grey diagonal line) reference strategies ​over a threshold probability range of approximately 0.06 to 0.90. This indicates enhanced clinical utility for the model across this threshold range.

### Prediction model performance in the Temporal validation cohort

**Fig. 6:**
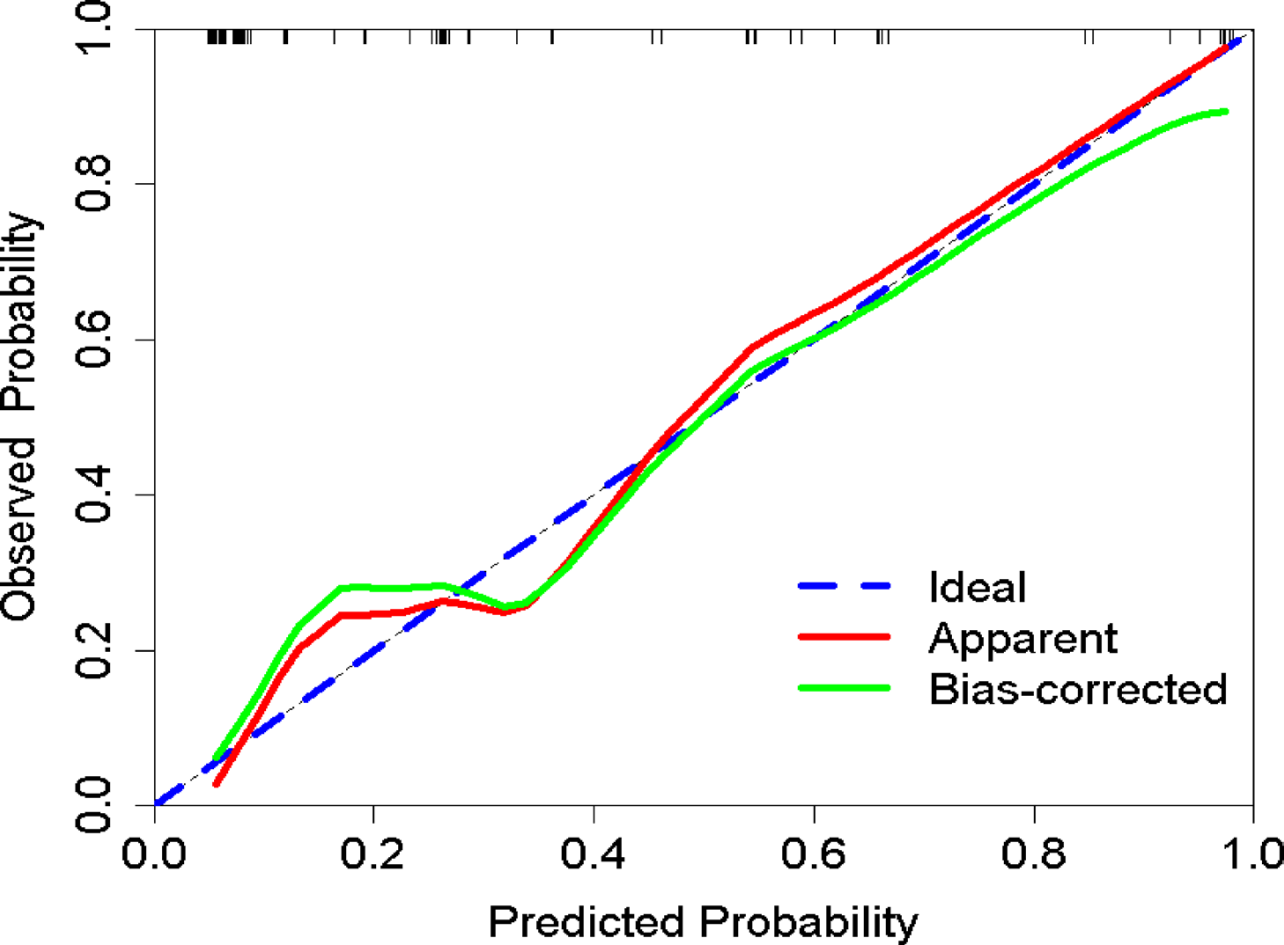
Calibration curve of the nutritional risk prediction model in the temporal validation cohort.

**Fig. 7:**
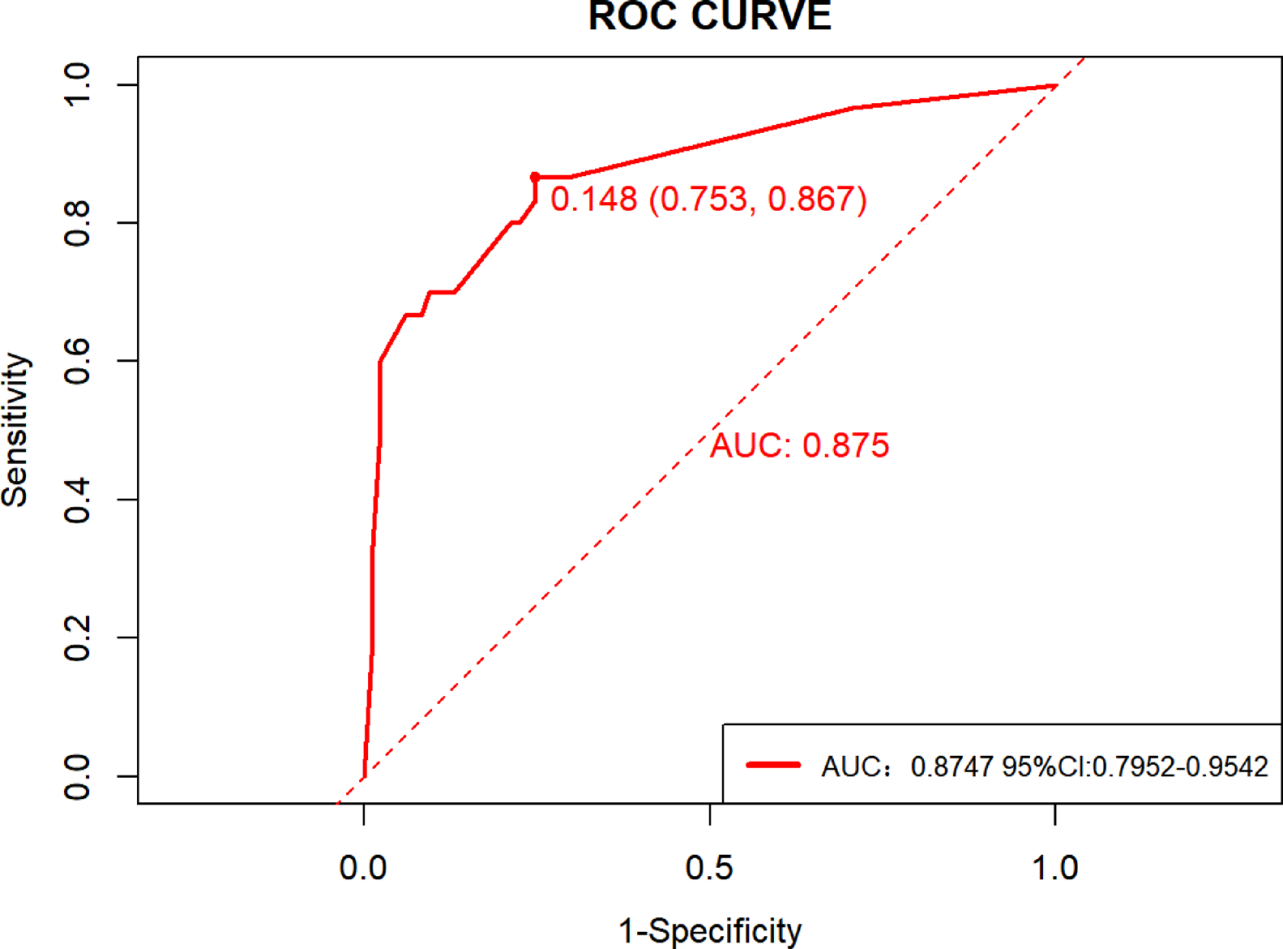
Receiver operating characteristic (ROC) curve of the model in the temporal validation cohort (AUC = 0.875, 95% CI: 0.795–0.954).

**Fig. 8:**
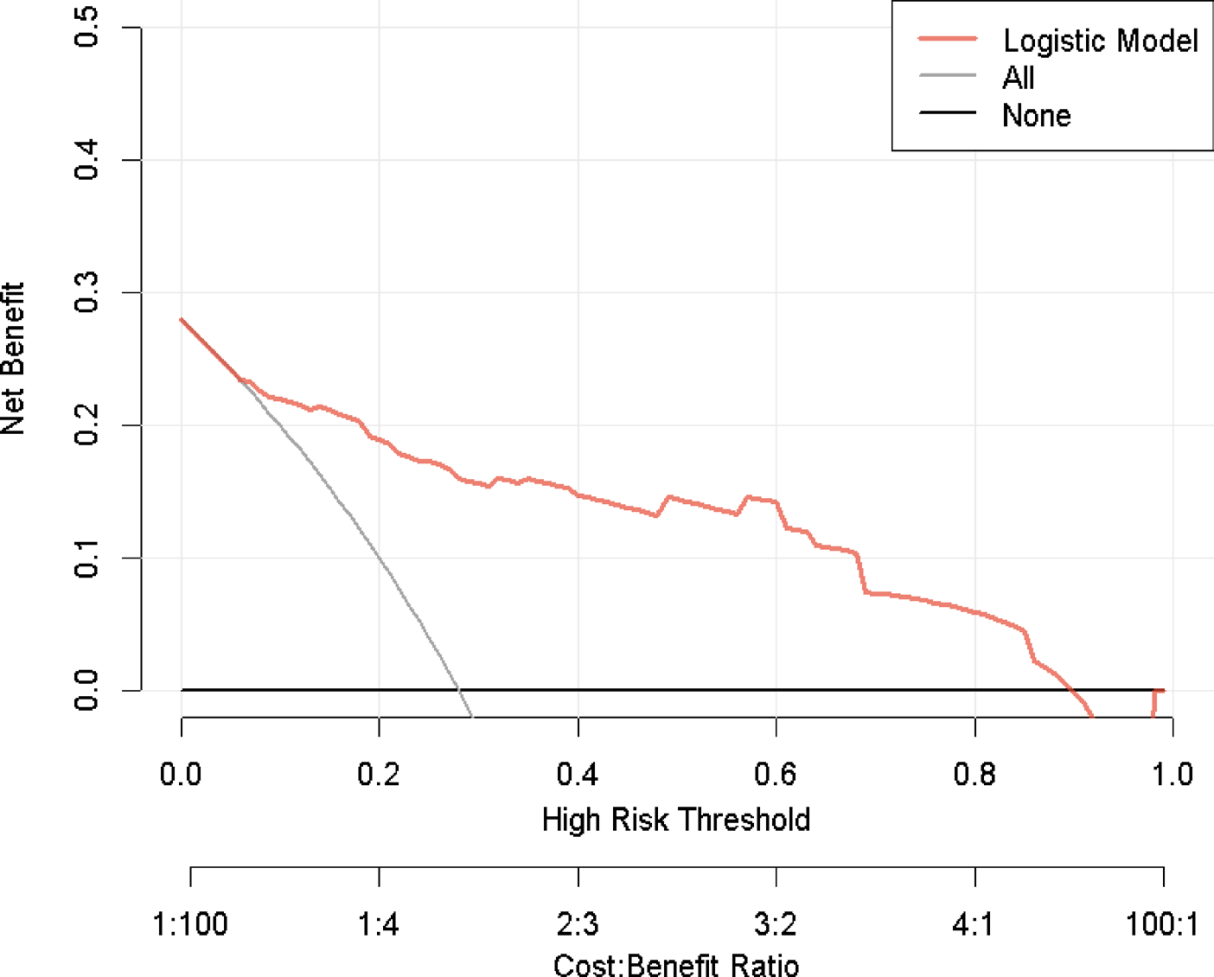
Decision curve analysis (DCA) of the model in the temporal validation cohort, demonstrating sustained clinical utility.

The calibration curve in Supplemental Fig. 3 demonstrates favorable overall model performance with a Brier score of 0.108 (< 0.25), slope (Z value) of − 0.157, and a nonsignificant P-value of 0.875 (> 0.05). Figure 6 further confirms strong agreement between predicted and observed probabilities, indicating excellent calibration capability of the prediction model. temporal validation results (Fig. 7) show an area under the ROC curve (AUC) of 0.8747 (95% CI: 0.7952–0.9542), with an optimal risk cutoff of 0.148 yielding specificity of 86.7%, sensitivity of 75.3%, accuracy of 0.7826, and precision of 0.5532. The decision curve analysis (DCA, Fig. 8) reveals enhanced clinical utility across threshold probabilities of 0.06 to 0.92, as evidenced by the model’s red curve being superior to both the “treat none” (black horizontal line) and “treat all” (light grey diagonal line) strategies.

## Discussion

This study developed and validated a UC-specific nutritional risk prediction model using five routinely available clinical parameters. The model demonstrated strong predictive performance, with excellent discrimination in both derivation and temporal validation cohorts, along with good calibration and clinical utility across a wide risk threshold range.

The key advantages of our model are its disease-specific design and its capacity for proactive risk prediction. Unlike general screening tools such as NRS-2002, our model incorporates UC-relevant predictors, particularly detailed disease activity, which is a well-established driver of nutritional deterioration^20^. Furthermore, it leverages these routine parameters to predict the outcome of an NRS-2002 screening (a score ≥ 3). This approach complements rather than replaces standard screening by enabling early, chart-based risk identification, potentially before a formal NRS-2002 is performed. This capability is ideally suited for integration into gastroenterology workflows, providing real-time decision support without adding to clinical workload, thereby streamlining the process for clinicians^6,19^. For the scenario of hospital admission, our model enables a clear clinical workflow: upon admission, a clinician (or an automated EMR system) can input the five readily available parameters. The resulting risk probability can then triage patients. Those at high risk (e.g., a nomogram score ≥ 180, corresponding to > 70% probability) can be flagged for immediate dietitian referral and nutritional support, while those at moderate risk can receive targeted education and monitoring. With EMR integration, this risk calculation could be automated, providing real-time decision support without adding to clinical workload, and representing a practical shift from reactive screening to proactive, data-driven risk stratification.

While the predictors in our model have been individually linked to nutritional status in IBD^6,8,21,22^, this is the first study to synthesize them into a dedicated UC-specific prediction tool. The variable selection process, guided by rigorous multivariable analysis, excluded inflammatory markers including CRP and ESR due to multicollinearity with disease activity (VIF > 5 for both markers), underscoring the importance of statistical refinement. The decision to retain hemoglobin, despite a borderline significance level (*p* = 0.065), was supported by sensitivity analysis confirming its consistent contribution to model performance across datasets, in addition to its well-established clinical relevance in reflecting the interplay of chronic blood loss, malabsorption, and nutritional status in UC^23,24^.

Several limitations of our study should be acknowledged. First, and most importantly, the retrospective design precluded direct comparison of our model’s performance against established tools like NRS-2002 or MUST, as only binary nutritional risk outcomes (NRS-2002 ≥ 3) were available rather than continuous scores required for comparative ROC analysis. Therefore, this study cannot assess whether the model offers a real advantage over these standard methods. Future prospective studies that administer multiple screening tools in parallel are essential to evaluate the comparative and added value of this UC-specific approach. Second, our model does not include classic nutritional indicators such as documented weight loss or prealbumin, as these were inconsistently available in our institutional records, potentially affecting its comprehensiveness. Third, the dietary variable, classified retrospectively from medical records, is susceptible to misclassification bias and variability in documentation standards compared to standardized prospective assessments, which could affect the robustness of this model input. Fourth, regarding model evaluation, while the model demonstrated good calibration and internal validation via bootstrap resampling showed minimal optimism, we did not apply formal shrinkage techniques. Future studies could consider such approaches to potentially enhance the model’s generalizability. Finally, the single-center origin and temporal (rather than fully external) validation of our model limit its immediate generalizability. Our findings should be considered preliminary and require confirmation in prospective, multi-center, and geographically diverse cohorts to establish broader clinical reliability.

## Conclusion

We present a practical, preliminary nutritional risk prediction model specifically for UC patients. Its clinical reliability across different healthcare settings must be established through future external validation studies. Future prospective studies should focus on implementing this model in clinical practice and evaluating its impact on hard patient outcomes, such as hospital readmission rates and surgical complications.

## Supplementary Information

Below is the link to the electronic supplementary material.

Supplementary Material 1

## Data Availability

The datasets generated during and/or analysed during the current study are available from the corresponding author on reasonable request.
