# Supplementary material for: Development and validation of a nutritional risk prediction model for patients with ulcerative colitis: a single-center retrospective study
Source: Sci Rep. 2025 Dec 29;16:3420. doi: 10.1038/s41598-025-33379-8 (PMC12834992; doi:10.1038/s41598-025-33379-8)
Supplement: Supplementary file 1 — Supplementary Material 1 [file 41598_2025_33379_MOESM1_ESM.docx]

**Table 1. Results of univariate analysis**

| Variables | No nutritional risk  （n=85） | Nutritional risk  （n=110） | P |
| --- | --- | --- | --- |
| Gender（n%） |  |  | 0.064 |
| Male | 70（54.26） | 205（63.66） |  |
| Female | 59（45.74） | 117（36.34） |  |
| Age at onset (years)（n%）​ |  |  | 0.079 |
| ≤16 | 2（1.55） | 0 |  |
| 17~40 | 57（44.19） | 149（46.27） |  |
| ＞40 | 70（54.26） | 173（53.73） |  |
| Marital status（n%） |  |  | 0.206 |
| Unmarried | 14（10.85） | 23（7.14） |  |
| Married | 112（86.82） | 296（91.93） |  |
| Other | 3（2.33） | 3（0.93） |  |
| Payment Method for Medical Expenses （n%） |  |  | 0.586 |
| Urban Employee | 41（31.78） | 99（30.75） |  |
| Urban Resident | 67（51.94） | 157（48.75） |  |
| Other | 21（16.28） | 66（20.50） |  |
| Disease duration (years)（n%） |  |  | 0.023 |
| ≤5 | 97（75.20） | 211（65.53） |  |
| 6~10 | 14（10.85） | 71（22.05） |  |
| ＞10 | 18（13.95） | 40（12.42） |  |
| Hospital stay (days)（n%） |  |  | <0.001 |
| ≤8 | 56（43.41） | 218（67.70） |  |
| >8 | 73（56.59） | 104（32.30） |  |
| Smoking history（n%） |  |  | 0.293 |
| NO | 115（89.15） | 275（85.40） |  |
| Yes | 14（10.85） | 47（14.60） |  |
| History of alcoholism（n%） |  |  | 0.618 |
| NO | 120（93.02） | 295（91.61） |  |
| Yes | 9（6.98） | 27（8.39） |  |
| Treatment Regimen（n%） |  |  | <0.001 |
| Monotherapy | 84（65.12） | 280（86.96） |  |
| Polytherapy | 45（34.88） | 42（13.04） |  |
| Diabetes（n%） |  |  | 0.044 |
| NO | 128（99.22） | 307（95.34） |  |
| Yes | 1（0.78） | 15（4.66） |  |
| Sleep Status（n%） |  |  | <0.001 |
| Normal | 65（50.39） | 246（76.40） |  |
| Abnormal | 64（49.61） | 76（23.60） |  |
| Diet（n%） |  |  | <0.001 |
| Normal | 53（41.09） | 266（82.61） |  |
| Abnormal | 76（58.91） | 56（17.39） |  |
| BMI（n%） |  |  | <0.001 |
| Underweight | 85（65.89） | 3（0.93） |  |
| Normal | 43（33.33） | 224（69.57） |  |
| Overweight | 1（0.78） | 76（23.60） |  |
| Obese | 0 | 19（5.90） |  |
| Educational level（n%） |  |  | 0.619 |
| Primary and below | 44（34.11） | 104（32.30） |  |
| Middle and high school | 45（34.88） | 128（39.75） |  |
| College and above | 40（31.01） | 90（27.95） |  |
| Disease Activity（n%） |  |  | <0.001 |
| Remission period | 1（0.77） | 51（15.84） |  |
| Mild | 4（3.10） | 69（21.43） |  |
| Moderate | 17（13.18） | 167（51.86） |  |
| Severe | 107（82.95） | 35（10.87） |  |
| Type of Lesion（n%） |  |  | 0.327 |
| Initial Onset | 5（3.88） | 20（6.21） |  |
| Chronic Relapsing Type | 124（96.12） | 302（93.79） |  |
| ​Extent of Lesion（n%） |  |  | <0.001 |
| ​Rectal Type | 8（6.20） | 81（25.16） |  |
| Left-Sided Colonic Type | 24（18.60） | 110（34.16） |  |
| Extensive Colonic Type | 97（75.20） | 131（40.68） |  |
| Albumin（n%） |  |  | <0.001 |
| Normal | 14（10.85） | 200（62.11） |  |
| Abnormal | 115（89.15） | 122（37.89） |  |
| Erythrocyte Sedimentation Rate（n%） |  |  | <0.001 |
| Normal | 15（11.63） | 82（25.47） |  |
| Abnormal | 114（88.37） | 240（74.53） |  |
| C-reactive protein（n%） |  |  | <0.001 |
| Normal | 40（31.01） | 242（75.16） |  |
| Abnormal | 89（68.99） | 80（24.84） |  |
| Hemoglobin（n%） |  |  | 0.002 |
| Normal | 18（13.95） | 99（30.75） |  |
| Abnormal | 111（86.05） | 223（69.25） |  |
| Complications（n%） |  |  | 0.866 |
| NO | 125（96.90） | 311（96.58） |  |
| Yes | 4（3.10） | 11（3.42） |  |
| History of gastrointestinal surgery（n%） |  |  | 0.645 |
| NO | 118（91.47） | 290（90.06） |  |
| Yes | 11（8.53） | 32（9.94） |  |

**Table 2. Collinearity Diagnostics of Independent Variables**

| **Variable** | **Tolerance** | **Variance Inflation Factor (VIF)** |
| --- | --- | --- |
| Hospitalization duration | 0.789 | 1.2675 |
| Payment method | 0.775 | 1.291 |
| Marital status | 0.864 | 1.157 |
| Age at onset | 0.768 | 1.302 |
| Disease duration | 0.880 | 1.136 |
| Smoking | 0.656 | 1.524 |
| Alcohol use | 0.691 | 1.448 |
| Treatment regimen | 0.881 | 1.135 |
| Diabetes | 0.927 | 1.079 |
| Sleep status | 0.762 | 1.312 |
| Dietary pattern | 0.683 | 1.463 |
| Education level | 0.735 | 1.361 |
| BMI category | 0.735 | 1.361 |
| Sex | 0.337 | 2.972 |
| Disease activity | 0.499 | 2.001 |
| Type of lesion | 0.942 | 1.062 |
| Extent of lesion | 0.693 | 1.443 |
| Albumin | 0.606 | 1.650 |
| Erythrocyte sedimentation rate | 0.415 | 2.412 |
| C-reactive protein | 0.605 | 1.652 |
| Hemoglobin | 0.330 | 3.029 |
| Complications | 0.949 | 1.054 |
| History of GI surgery | 0.961 | 1.040 |


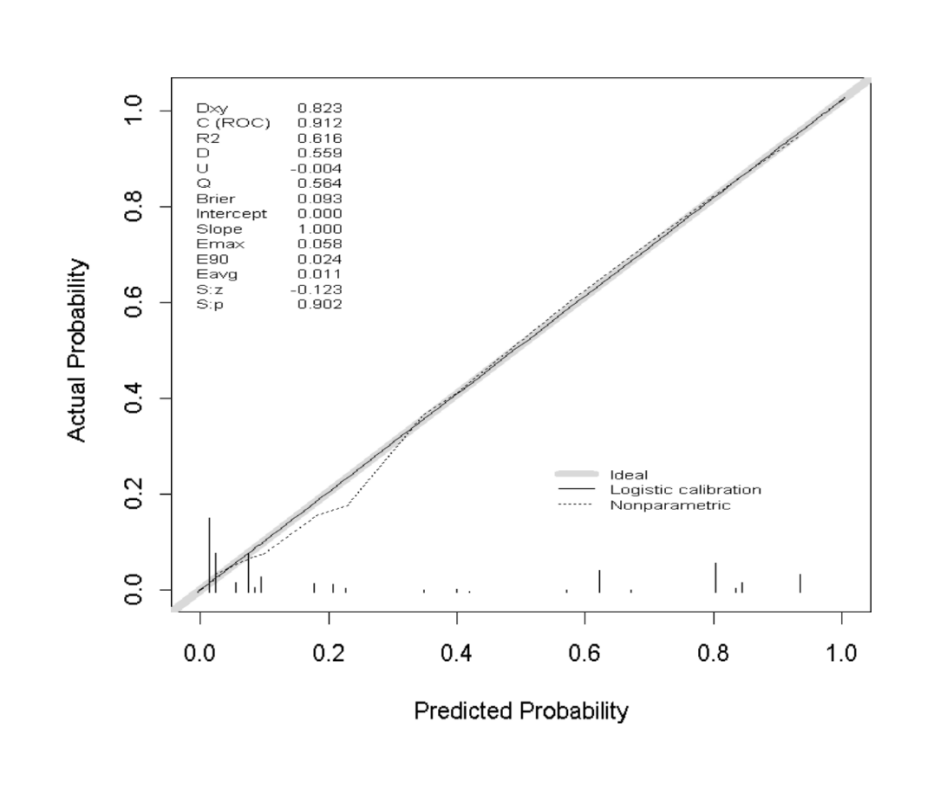


**Figure 1.** Calibration curve of the nutritional risk prediction model in the Derivation cohort

**Table 3.** Confusion Matrix for UC Nutritional Risk Prediction in the Derivation Cohort

|  | Actual Nutritional Risk (Positive) | Actual No Nutritional Risk (Negative) |
| --- | --- | --- |
| Predicted Nutrition Risk (Positive) | TP: 109 | FP: 31 |
| Predicted No Nutrition Risk (Negative) | FN: 20 | TN: 291 |


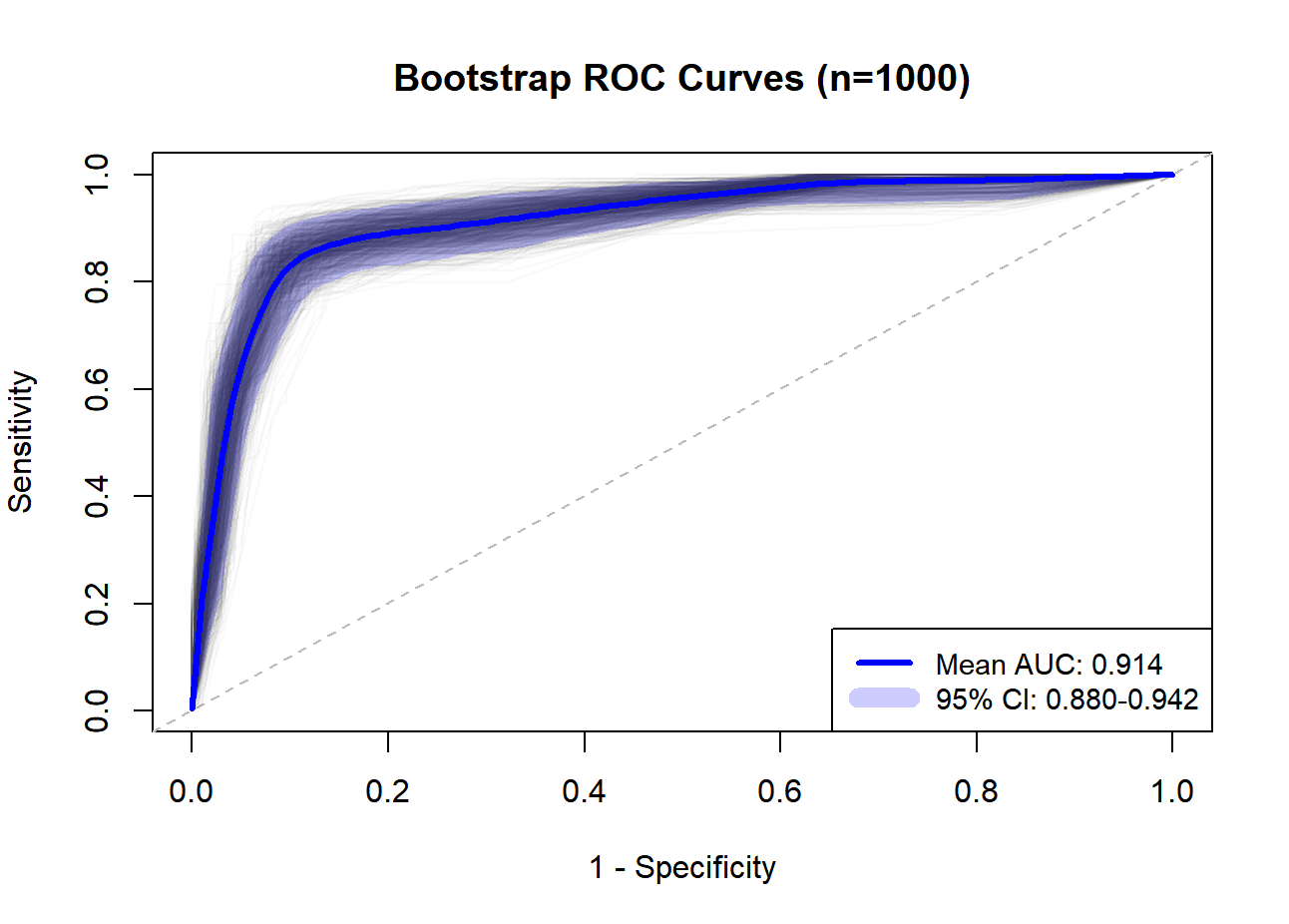
**Figure 2.** Bootstrap-Resampled ROC Curve of the Nutritional Risk Prediction Model in the Derivation Cohort


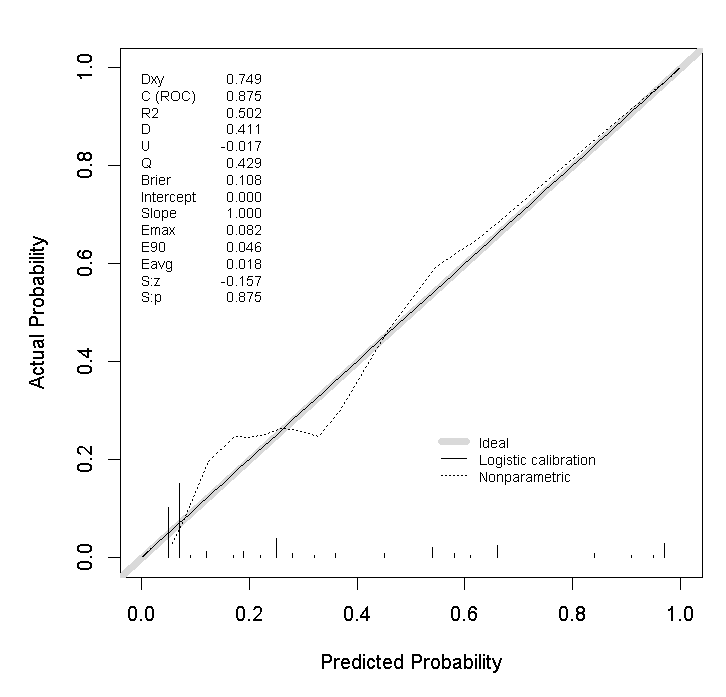


**Figure 3.** Calibration curve of the nutritional risk prediction model in the Validation Cohort
